# Prevalence of Type 2 Diabetes Mellitus in Adult Population of Pakistan: A Meta-Analysis of Prospective Cross-Sectional Surveys

**DOI:** 10.5334/aogh.2679

**Published:** 2020-01-31

**Authors:** Muhammad Adnan, Muhammad Aasim

**Affiliations:** 1 Pakistan Health Research Council (PHRC) Research Center, Fatima Jinnah Medical University, Lahore, PK; 2 National Health Research Complex, PHRC Research Center, Shaikh Zayed Hospital, Lahore, PK

## Abstract

**Background:**

The clinical and methodological diversity observed in national and regional diabetes surveys, emphasized on the need of the weighted average prevalence of diabetes.

**Objective:**

To measure the pooled prevalence of type 2 diabetes mellitus in the adult population of Pakistan.

**Methods:**

The prospective cross-sectional studies reporting adult diabetes in Pakistan and published on any date were retrieved from PubMed, ScienceDirect and PakMediNet databases. In the meta-analysis, PRISMA guidelines were used for reporting; the AXIS tool for assessing quality and risk of bias within studies; I^2^ statistics for measuring heterogeneity between studies and subgroups; and Tableau Public 10.4 for geographic mapping of included studies. Using Meta-Analyst 3.13 βeta, overall and subgroup pooled estimates were measured by random effects model.

**Results:**

The pooled sample of twelve studies included 42,051 adults (≥20 years) comprised of both sexes from urban and rural Pakistan. The pooled prevalence of diabetes was 13.7% (95% CI, 10.7–17.3). None of the twelve studies was of poor quality (<10 scores). Ten studies were published in ISI indexed journals, and nine of them were indexed for Medline. The level of heterogeneity observed across studies and between subgroups was moderate (<50%). The subgroup analysis revealed a higher pooled estimate of diabetes in males than in females (13.1 vs. 12.4%). It was also higher in urban than in rural patients (15.1 vs. 1.6%), and in HbA1c than in OGTT tests (23.9 vs. 14.4%). However, pooled estimates of the WHO and the ADA criteria were similar (13.8 vs. 13.5%).

**Conclusions:**

The prevalence of diabetes is on the rise in the adult population of Pakistan. The heterogeneity across studies observed in the meta-analysis suggested that the design of future diabetes surveys should be efficient and purposeful, and that valid tools and methods should be used to generate more precise data. Moreover, harmony between the stakeholders is much needed to seek a true picture of the diabetes burden in the country.

## Introduction

Diabetes mellitus (DM) has a strong influence on the quality and length of patients’ lives, and puts a significant financial burden on them [1,2]. The International Diabetes Federation (IDF) diabetes atlas 2017 ranks Pakistan at 10 of 221 countries of the world, having 7.5 million cases of diabetes (20–79 years) [3]. Moreover, the World Health Organization (WHO) diabetes country profiles 2016 state that the national response to diabetes—including operational policy, national diabetes guidelines and diabetes registry—is not available in Pakistan [4]. However, three national diabetes surveys have been conducted in Pakistan since 1947.

The first national diabetes survey of Pakistan (NDSP-I) was accomplished in four phases (1995–98), and the combined data were published in 2007. The total number of subjects (n = 5433) in NDSP-I combined data was higher than the sum of subjects examined in four individual studies [5,6,7,8,9]. Therefore, the prevalence of diabetes estimated in NDSP-I was probably under-reported. The second national diabetes survey of Pakistan (NDSP-II) was conducted in 2016–17, and the third diabetes prevalence survey of Pakistan (DPS-PAK) was conducted in 2017 [10,11]. Though NDSP-II and DPS-PAK were conducted simultaneously, the respective prevalence rates were markedly different (26.3 vs.16.98%). In the last two decades, some regional diabetes surveys had also been conducted in Pakistan, but the reported prevalence of diabetes varied between 0.95% and 32.9% [12,13,14,15,16,17,18,19,20,21,22,23,24].

The inter-studies variations observed in the prevalence of diabetes, resulting from differences in the characteristics of subjects, screening tools, diagnostic criteria, and settings of the studies, emphasized the need of a weighted average prevalence of diabetes. Therefore, the current meta-analysis was aimed to measure the pooled prevalence of type 2 diabetes mellitus (T2DM) in the adult population of Pakistan.

## Methods

### Protocol and Registration

The meta-analysis had no documented protocol. However, the preferred reporting items for systematic reviews and meta-analyses (PRISMA) guidelines were used for reporting [25].

### Eligibility Criteria

In view of the main outcome—adult diabetes in Pakistan—the criteria of eligible studies included prospective, population-based, cross-sectional diabetes surveys. Eligible works were published as original research articles, in the English language, on any date, and in any journal. Similarly, the criteria of eligible subjects included adults (≥18 years) of both sexes belonging to any socio-economic class, living in any area of Pakistan.

### Information Sources and Search Process

The databases such as PubMed, ScienceDirect and PakMediNet were searched for eligible records between October 2018 and March 2019. The MeSH keywords “prevalence”, “diabetes” and the term “national diabetes survey of Pakistan” were entered in the advanced search option of PubMed to identify the eligible records. The identified records were further refined by using the following filters: species human, language English, journals Medline, age adult 19^+^ years, and search field title/abstract. The filtered records were sorted by best match, and the titles of studies were evaluated for relatedness. The relevant studies were selected and exported to the summary text file for further assessment. Similar searches were made on the ScienceDirect and PakMediNet databases. Additionally, a few records were identified from the references of other studies by performing a manual search.

### Screening of Abstracts and Full-Text Articles

The abstracts of relevant studies were screened using the criteria for eligible studies. The following studies were excluded: hospital-based studies, clinic-based studies, medical camp-based studies and studies involving subjects with a specific illness. Then, full-text articles were screened using the criteria for eligible studies/subjects, and a few more studies were excluded. These included studies involving both adult and adolescent populations, studies of the geriatric population, studies of the high-risk population, studies not reporting screening tool and diagnostic criteria, and studies reporting combined data of selected individual studies.

### Quality Assessment

An appraisal tool for cross-sectional studies (AXIS) was used to assess the risk of bias, quality of design, and quality of reporting of selected full-text articles [26]. The tool had 20 questions, and each question had three responses (yes, no & don’t know), increasing the score by one for each yes. Each individual study received a score between 0 and 20. Based on these scores, the individual studies were categorized into three groups: Good (>15), fair (10–15) & poor (<10). Additional to AXIS quality assessment, the index of journals those published the twelve included studies had been evaluated.

### Data Items and Collection Process

The study variables included the last name of the first author, year of publication, study design, settings, screening tool, diagnostic criteria, participants’ characteristics, sample size, numbers of males and females, numbers of positive cases, and the journal’s name and index. The data were retrieved and extracted by the first author. However, the process was repeated multiple times. The authors of included studies were not approached for the sake of data.

### Data Analysis

Using the pre-assigned unique codes, the data were entered in a Microsoft Excel worksheet. The extracted data were neither combined nor transformed. The Meta-Analyst version 3.13 βeta was used for the quantitative analysis [27]. The I^2^ statistic was used to measure the heterogeneity across studies and between subgroups, and the I^2^ values of 25%, 50%, and 75% were considered as low, moderate and high, respectively [28]. The overall and subgroup pooled prevalence percentages of DM were measured using a random effects model [29]. Notably, the subgroup pooled estimate of males and females included eleven out of twelve studies. The cumulative estimate was presented by using a forest plot, and the subgroup pooled estimates were presented by the combination of bar and line charts. Tableau Public 10.4 was used for the geographic mapping of districts surveyed in the included studies [30].

## Results

### Selection Process of Eligible Studies

**Figure 1:**
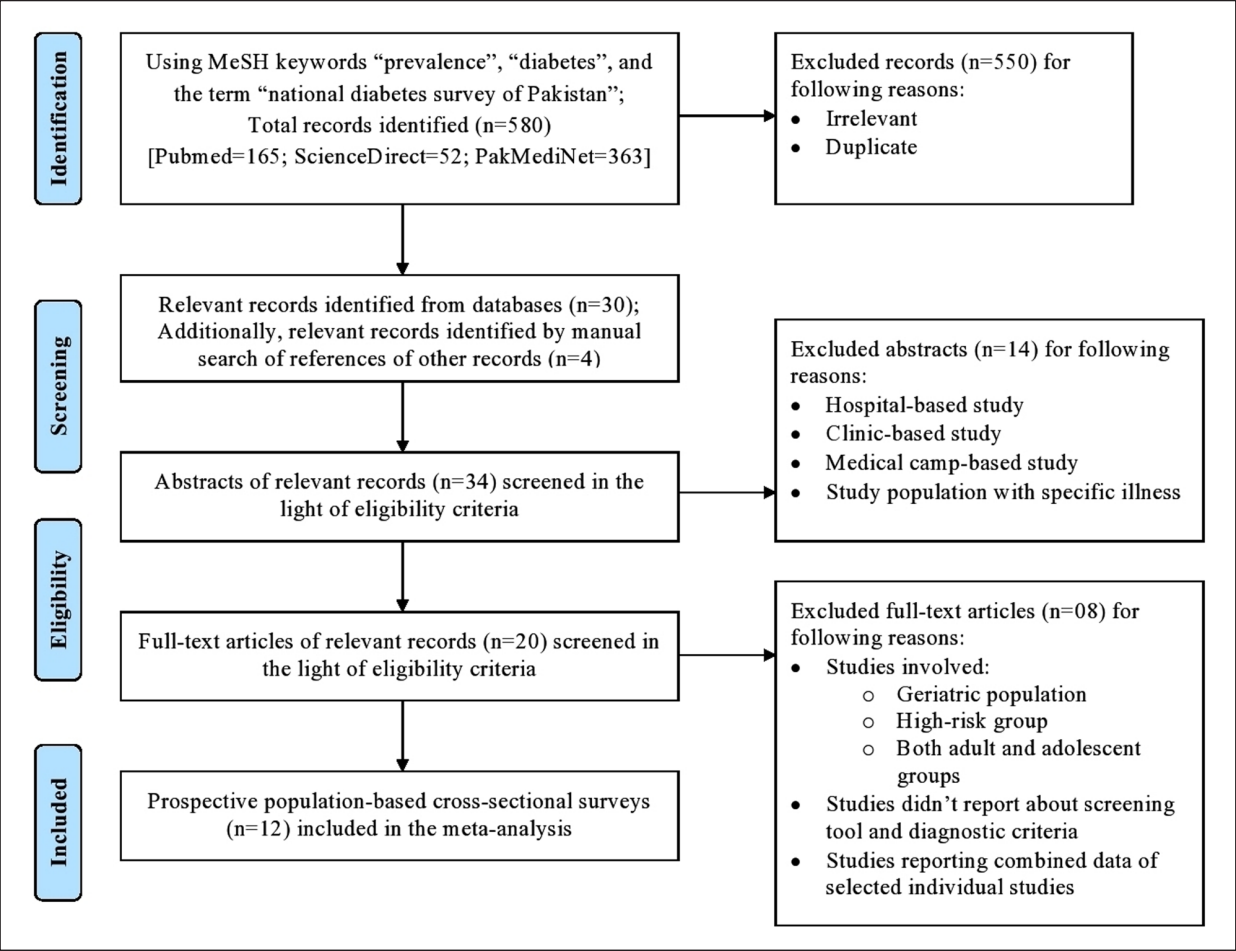
PRISMA flow diagram showing selection process of included studies.

A total of 580 records were identified from three databases. Twelve studies, including two national and ten regional diabetes surveys, met the eligibility criteria and were selected for quantitative analysis. The PRISMA flow diagram was used to present the process of selecting studies (Figure 1).

### Characteristics and Results of Included Studies

All included studies were published between 1995 and 2019 and in the English language. The design of the studies was a prospective cross-sectional survey. The sample size across studies ranged between 404 and 18,856 subjects. Using a probability sampling technique, all studies enrolled adults (≥20 years) of both sexes living in Pakistan. The participation of females was higher than of males. Both urban and rural populations were included in five studies, only the urban population in two studies, and only the rural population in five studies. OGTT was used as the diabetes screening tool in five studies, with BSF used in four studies, HbA1c in two studies, and BSR in one study. The WHO diagnostic criteria were used in eight studies and ADA criteria in four studies. The prevalence of diabetes across studies ranged between 6.3% and 32.9% (Table 1).

**Table 1:** Baseline characteristics of selected national and regional diabetes surveys.

Author & Year	Sample size (n)	Age (years)	M:F	Screening tool	Diagnostic criteria	Prevalence (%)	ISI-indexed Journal	Quality score	Weight assigned

Shera et al. (1995) [6]	967	≥25	1:1.49	OGTT	WHO	13.50	Yes	13/20	2.305
Shera et al. (1999) [7]	1404	≥25	1:2.22	OGTT	WHO	13.40	Yes	13/20	3.328
Shera et al. (1999) [8]	1035	≥25	1:4.00	OGTT	WHO	11.10	Yes	11/20	2.080
Basit et al. (2002) [12]	2032	≥25	1:2.03	BSF	ADA	6.30	Yes	10/20	2.423
Shera et al. (2010) [9]	1852	≥25	1:1.27	OGTT	WHO	10.80	Yes	15/20	3.646
Basit et al. (2011) [13]	1264	≥25	1:1.98	BSF	ADA	14.20	Yes	12/20	3.127
Ahmad et al. (2011) [14]	938	≥30	1:1.31	BSR	WHO	10.90	No	11/20	1.850
Qureshi et al. (2014) [15]	815	≥20	1:1.41	BSF	ADA	9.93	No	18/20	1.484
Zafar et al. (2016) [16]	404	≥20	1:1.23	HbA1c	ADA	32.90	Yes	16/20	1.815
Akhtar et al. (2016) [17]	1650	≥20	1:0.96	BSF	WHO	11.10	Yes	14/20	3.311
Basit et al. (2018) [10]	10834	≥20	1:1.27	OGTT	WHO	26.30	Yes	18/20	42.652
Aamir et al. (2019) [11]	18856	≥20	1:0.86	HbA1c	WHO	16.98	Yes	19/20	54.080

Abbreviations: M: Male; F: Female; HbA1c: Glycated hemoglobin; OGTT: Oral Glucose Tolerance Test; BSR; Blood Sugar Random; BSF: Blood Sugar Fasting; WHO: World Health Organization; ADA: American Diabetes Association.

### Geographic Mapping of Surveyed Districts

**Figure 2:**
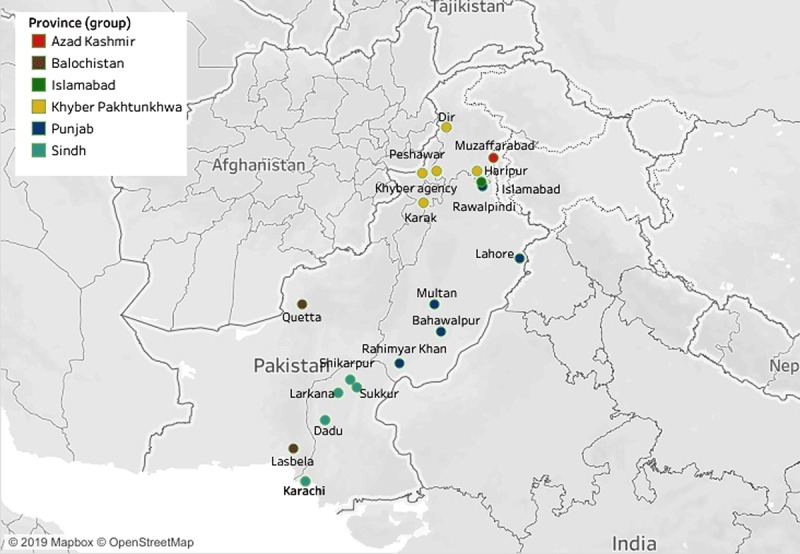
Geographic mapping of the districts been surveyed.

A total of nineteen districts had been surveyed at least once in twelve selected studies. In Figure 2, the geographic mapping shows that five districts each of Punjab (PB), Sindh (SD) and Khyber Pakhtunkhwa (KP) were included in the surveys. Other areas surveyed included two districts of Balochistan (BA), one district of Azad Jammu & Kashmir (AJK), and one of Islamabad Capital Territory (ICT).

### Quality/Risk of Bias

All included studies had a cross-sectional design, followed probability sampling, enrolled subjects from the general adult population of Pakistan, and reported prevalence of diabetes. Therefore, the process of selecting studies reduced the heterogeneity to a negligible level. The evaluation of journals’ index publishing of the individual studies showed that 10 studies were published in ISI indexed journals, and that nine of them were indexed for Medline. The AXIS quality assessment revealed that none of the 12 studies had received scores <10 (poor), eight studies had scores 10–15 (fair), and four studies had scores >15 (good). The level of heterogeneity observed across studies and between subgroups was moderate (<50%).

### Synthesis of Results

**Figure 3:**
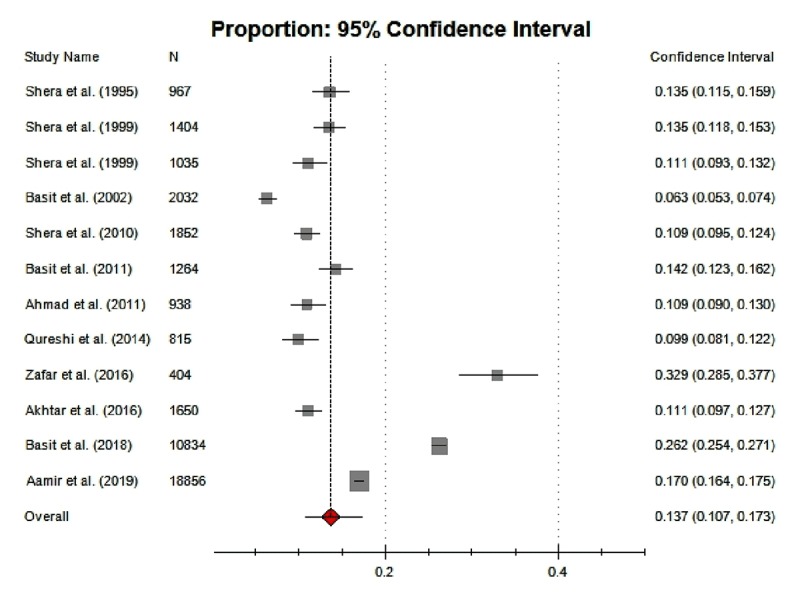
Pooled prevalence of T2DM in adult population of Pakistan.

The pooled sample of twelve studies included 42,051 adults (≥20 years) of both sexes from urban and rural Pakistan. The pooled prevalence of adult diabetes was 13.7% (95% CI, 10.7–17.3) in Pakistan. The weight assigned to individual studies ranged between 1.484 and 54.080. As shown in Figure 3, the pooled estimate of diabetes obtained in the meta-analysis was higher than the prevalence reported by six individual studies, and was lower than that of three studies.

### Additional Analysis

**Figure 4:**
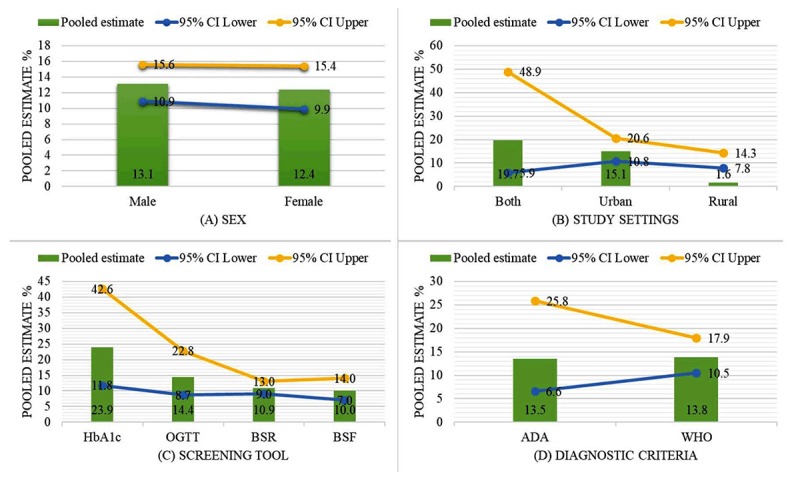
Comparison between subgroups pooled estimates.

The comparison between subgroups showed that males had a higher pooled estimate of diabetes than did females (13.1 vs. 12.4%) (Figure 4a). The studies including both urban and rural populations had the highest pooled estimate of diabetes (19.7%), followed by the urban (15.1%) and the rural (1.6%) populations alone (Figure 4b). The studies using HbA1c as the diabetes screening tool had the highest pooled estimate (23.9%), followed by OGTT (14.4%), BSR (10.9%) and BSF (10.0%) (Figure 4c). However, the studies using the WHO diagnostic criteria had a similar pooled estimate as those of the ADA criteria (13.8 vs. 13.5%) (Figure 4d).

## Discussion

The IDF diabetes atlas 2017 ranks Pakistan at number 2 out of 21 countries for the prevalence of diabetes in the Middle East and North Africa (MENA) region, with 7.5 million cases of diabetes (20–79 years), and at number 18 out of 21 countries for its 6.9% prevalence of diabetes (20–79 years) [3]. Pakistan has 27.4 million cases of diabetes (≥20 years), estimated from the prevalence of diabetes at 26.3% by the NDSP-II 2016–17 [10,31]. Differently, the pooled prevalence of diabetes at 13.7% proposes that Pakistan has 13.8 million cases of adult diabetes (≥20 years). These pooled cases are twice higher than cases reported by the IDF; almost half of the cases estimated from the NDSP-II; yet close to 17.1 million cases of diabetes (≥20 years) derived from prevalence of diabetes 16.98% by the DPS-PAK 2017 [11].

It has been calculated from the provisional results of the 6^th^ population and housing census 2017 of Pakistan, excluding AJK and GB (10 districts each), that approximately 100.78 million adults (48.51% of a total 207.77 million) live in the ICT and 129 districts of Pakistan [32]. However, the pooled sample of current meta-analysis show that only 42,051 (0.042 million) adults from 19 districts of Pakistan have been examined in the 12 included studies. Furthermore, out of three national diabetes surveys conducted in four provinces, only the DPS-PAK included samples from the AJK, GB, and FATA regions.

More than 50% of the world’s population, and about 66.6% of the total diabetic population, live in urban areas [33]. Similarly, about 40.03 million adults (≥20 years) live in urban areas of Pakistan, and the overall population density has increased from 261 to 4000 people per square kilometer in big cities [32,34]. Besides the fact demonstrated in the geographic mapping, that the majority of diabetes surveys were conducted in or near big cities, the subgroup analysis validates that the urban population had a markedly higher pooled estimate of diabetes than that of the rural population.

DM affects both sexes almost equally, so a higher prevalence of diabetes has been observed in both males (18–74 years) and females (75–99 years), worldwide [33]. Differently, females have a higher prevalence of diabetes than males in all age groups in the MENA region [35]. Contrary to these facts, the subgroup analysis reveals that, in Pakistan, males have a higher pooled estimate of diabetes than females.

The studies prefer using OGTT as a diabetes screening tool over HbA1c in high-risk populations, and recommend that OGTT should be used to diagnose diabetes in patients with HbA1c 5.6–6.4%, because HbA1c may miss such cases [36,37]. Conversely, the studies using HbA1c have a higher pooled estimate of diabetes than do those using OGTT in the present study. The finding of similar pooled estimates in the general adult population by both the WHO diagnostic criteria of diabetes and the ADA criteria supports the evidence of disagreement between the criteria among obese and elder subjects [38,39].

It has been estimated from NDSP-II 2017 results that Pakistan has 27.4 million cases of diabetes [10,31]. In the same way, it is calculated from the 8.7% prevalence of diabetes by NDSP-I 1998 and 46.22% proportion of adults by the population census 1998 that approximately 5.32 million Pakistani adults had diabetes in 1998 [5,40]. The comparison between estimates of NDSP-I and II reveals a 415.03% increase in cases of diabetes, from 5.32 to 27.4 million. This eye-opening growth rate evidences that diabetes has become an epidemic in the country.

### Strengths and Limitations

The meta-analysis had no documented protocol. However, reporting was done using the PRISMA guidelines. None of the 12 studies were of poor quality (<10 scores). Ten studies were published in ISI indexed journals, and nine of them were indexed for Medline. The level of heterogeneity was moderate (<50%). The data were retrieved and extracted by the first author. However, the process was repeated multiple times. The authors of selected studies were not approached for the sake of data, and the extracted data were neither combined nor transformed. Additional analysis included the geographic mapping of the districts surveyed.

## Conclusion

The prevalence of diabetes is on the rise in the adult population of Pakistan. The heterogeneity across studies observed in the meta-analysis suggested that the design of future diabetes surveys should be efficient and purposeful, and that valid tools and methods should be used to generate more precise data. Moreover, harmony between the stakeholders is much needed to seek a true picture of the diabetes burden in the country.
